# Multiple differences in calling songs and other traits between solitary and gregarious Mormon crickets from allopatric mtDNA clades

**DOI:** 10.1186/1471-2148-7-5

**Published:** 2007-01-22

**Authors:** Nathan W Bailey, Darryl T Gwynne, William V Bailey, Michael G Ritchie

**Affiliations:** 1 Department of Biology, Spieth Hall, University of California, Riverside, CA 92521, USA; 2 Biology Department, University of Toronto at Missisauga, Missisauga, Ontario L5L 1C6, Canada; 3 School of Biology, University of St. Andrews, St. Andrews, Fife KY16 9TH, UK

## Abstract

**Background:**

In acoustic species, traits such as male calling song are likely to diverge quickly between allopatric populations due to sexual selection, and divergence in parameters such as carrier frequency, chirp structure, and other important song characters can influence sexual isolation. Here we make use of two forms of Mormon crickets to examine differences in a broad suite of traits that have the potential to influence speciation via sexual isolation. Mormon crickets in "gregarious" populations aggregate into dense migratory bands, and females are the sexually competitive sex (sex-role reversal). There is also a non-outbreak "solitary" form. These two forms are largely but not perfectly correlated with a significant mtDNA subdivision within the species that is thought to have arisen in allopatry. Combined information about multiple, independently evolving traits, such as morphology and structural and behavioural differences in calling song, provides greater resolution of the overall differences between these allopatric populations, and allows us to assess their stage of divergence. We test two predictions, first that the forms differ in song and second that gregarious males are more reluctant to sing than solitary males due to sex role reversal. We also tested for a difference in the relationship between the size of the forewing resonator, the mirror, and carrier frequency, as most models of sound production in crickets indicate that mirror size should predict carrier frequency.

**Results:**

Multivariate analyses showed that solitary and gregarious individuals from different populations representing the two mtDNA clades had almost non-overlapping distributions based on multiple song and morphological measurements. Carrier frequency differed between the two, and gregarious males were more reluctant to sing overall. Mirror size predicted carrier frequency; however, the relationship between mirror size and surface area varied between solitary and gregarious forms, suggesting that factors above and beyond mirror size contribute to carrier frequency.

**Conclusion:**

The two clades of Mormon crickets differ in a broad suite of independent traits that probably justify subspecies status (the two can successfully mate so may not be reproductively isolated). However, our results emphasize the importance of carefully distinguishing the ultimate causation of differences between traits used to delineate species or subspecies boundaries.

## Background

Sexually selected traits – such as male calling song – have been suggested to be particularly susceptible to divergence in allopatrically isolated populations [1], and sexual selection-induced differences in song have been proposed to be significant drivers of speciation [2-6]. Differences in male song may evolve particularly rapidly, sometimes even more rapidly than sexual isolation [1,7,8]. Regardless of the ultimate causation, divergence in such characters may be an important indicator of subspecies or incipient speciation events [6,7,9,10].

**Figure 1:**
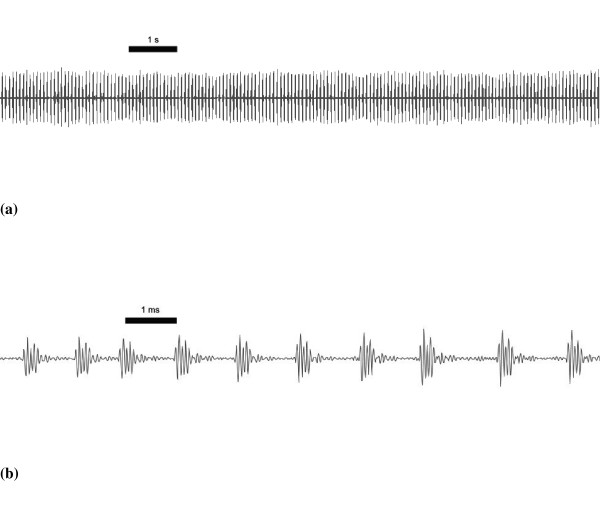
Oscillograms of Mormon cricket song showing **(a) **sustained singing and **(b) **damping of mirror oscillations between peg strikes.

The most common mechanism for singing in insects is stridulation, where specialised areas of the body are made to oscillate by striking against stridulatory pegs [11-13]. In katydids (or bushcrickets)(Tettigoniidae), the tegmina are modified so that during each wing-stroke, pegs on the stridulatory file of the upper (left) elytron strike the plectrum on the lower (right) elytron, causing the mirror, a circular membrane situated next to the plectrum, to vibrate [12-14]. In some ensiferan species, particularly Gryllidae, oscillation of the mirror (or harp in gryllids) is not damped between peg strikes and thus calls are "musical", or pure tone. However, many katydids, especially those found in the northern hemisphere, do not produce pure tone songs because resonance from each peg strike is highly damped before the onset of the next peg strike (see figure 1). Under the "clockwork cricket" model of stridulation developed from studies of *Gryllus campestris *[15,16], the dominant or carrier frequency of male song is determined by properties of the harp, and in tettigonids, the length of the mirror frame has been suggested to determine carrier frequency [17].

Mormon crickets (*Anabrus simplex*, Orthoptera, Tettigoniidae, Tettigoniinae) are flightless katydids ranging throughout the American west that occur in solitary and gregarious forms. Solitary individuals comprise all populations on the eastern slope of the Rocky Mountains and are cryptically coloured brown or green, whereas gregarious individuals are more widely distributed in the west, are typically black in colour and aggregate into destructive migratory bands that can travel up to 2 km per day [18,19]. Their banding behaviour reduces the risk of predation [20] and they may occur at densities over a thousand times higher than in solitary populations [21].

Solitary and gregarious Mormon crickets have different mating systems. Gwynne [22] demonstrated that Mormon crickets are sex-role reversed at high population density, with females competing for matings with discriminating males. Male tettigoniids transfer a spermatophylax (attached to the spermatophore) to females upon mating [23], and this "nuptial gift" represents an important food source for females when there is a greater proportion of sexually active females, as is the case when food availability is low in the dry sagebrush habitats where gregarious outbreaks occur [21,22,24,25]. Few studies have examined differences in male song in role-reversed ensiferans. Role-reversed males of the tettigoniid, *Metaballus litus*, produce a different type of song than typical males, and males from typical conditions facultatively change their song type when transferred to role-reversed sites [26]. In pollen feeding katydids *Kawanaphila nartee*, fewer males were observed singing in role-reversed populations that did not have access to high quality food [27]. The number of males and females available for mating, and thus mating roles and the proportion of singing males, can vary over relatively short geographical distances due to microhabitat differences between populations of this flightless species [27]. The proportion of calling males may also vary over time. For example, in the gryllid *Gryllus campestris*, more males adopted a calling strategy during the course of one breeding season when population density decreased and the sex ratio became increasingly skewed towards males [28]. In a tettigoniid, *Ephippiger ephippiger*, song traits vary under experimental conditions of role reversal [29]. Role-reversed males sang less frequently and at a lower intensity, but female preferences for one important song trait remained unchanged [29]. In natural populations of Mormon crickets, males at low-density (i.e. solitary) sites called for longer periods of time than those at high-density (i.e. gregarious) sites, mainly because females were attracted quickly in the latter sites [21], but neither reluctance to sing (the incidence of singing males) nor structural differences in song have previously been investigated.

Recent mtDNA studies have uncovered a significant genetic division within Mormon crickets that broadly corresponds to western gregarious populations and eastern solitary populations [31]. Molecular clock arguments suggest that this division is approximately 2 million years old, and the two lineages likely represent discrete evolutionary histories [31]. Role reversal and sexual selection during periods of allopatry have the potential to influence male calling song evolution and morphology, but here we do not attempt to disentangle the causative effects of these two processes. Instead, we examine song and morphological parameters to address two questions. First, do the two forms differ across a broad suite of traits? Second, are gregarious males more reluctant to sing than solitary males? A key prediction is that in gregarious populations, song parameters will reflect the consistently weaker sexual selection on males due to role-reversal plus selection to decrease the risk of attracting natural enemies attracted to song [23,32]. Following this prediction, we expect that gregarious males will be less likely to initiate singing, and that if they do sing, they will chirp more slowly and use less of their stridulatory file during each wing stroke (due to relaxed sexual selection). We also test whether carrier frequency (C_*f*_) and the allometric relationship between mirror size and carrier frequency differs between the forms. Finally, in line with Gwynne's [21] observation that sexual size dimorphism is more pronounced in gregarious populations (with females larger than males), relaxed sexual selection on gregarious males might be expected to increase their variance in body size relative to that of gregarious females and solitary males.

## Results and discussion

### Differences between forms

**Figure 2:**
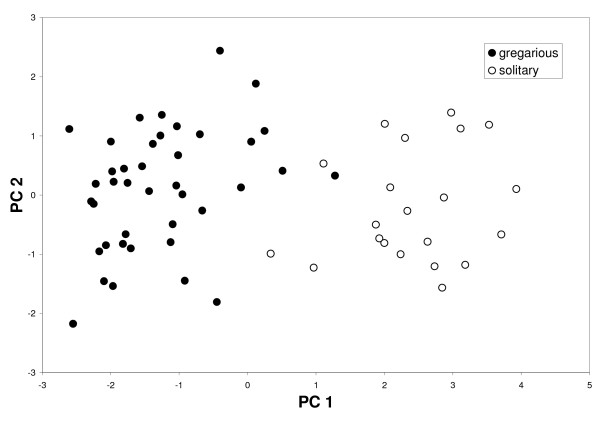
Solitary and gregarious Mormon crickets were almost completely distinguishable based on the first axis of a principal components analysis. As the first PC axis increases, HCW, elytral surface area and mirror surface area are smaller, whereas chirp rate, carrier frequency and peg strike rate are greater.

A principal components analysis almost completely distinguished the solitary (eastern clade) and gregarious (western clade) crickets (Figure 2). Most of the variation (89.00%) in the first principal components axis was between the two cricket forms (F_1,56 _= 218.89, P < 0.001) with only a small (0.72%) and non-significant (F_3,56 _= 1.84, NS) component amongst populations within forms. We therefore pooled populations into gregarious and solitary forms for further analyses. In a multivariate discriminant analysis, cross-validation successfully classified all samples into the correct form, and the traits with the highest loadings on the discriminant function were: head capsule width (HCW), elytral surface area and mirror surface area (which get smaller in the first principal components axis), and carrier frequency, chirp rate and peg strike rate (which get greater in the first principal components axis). Thus solitary males had smaller HCWs, smaller elytral surface area, smaller mirror surface areas, higher carrier frequencies, chirped faster and had more peg strikes per wing stroke (table 1). Temperature did not have an effect on chirp rate or the number of peg strikes per chirp, but solitary individuals chirped faster than gregarious ones (GLM: F_1,57 _= 139.28, P < 0.001) and used more stridulatory pegs for each wing stroke (GLM: F_1,57 _= 20.77, P < 0.001). Despite these differences in song structure, the number of stridulatory pegs did not differ between solitary and gregarious males (t-test: t_59 _= 1.24, NS) (table 1).

**Table 1:** Differences between solitary and gregarious populations in morphological and song measurements. Means are given with 95% confidence intervals, and those in bold indicate parameters that differed between forms.

	head capsule width (mm)	elytral surface area (mm^2^)	mirror surface area (mm^2^)	carrier frequency (kHz)	chirp rate (chirps/sec)	peg strikes per chirp	number of pegs on file
solitary	**6.71 **± 0.10	**26.88 **± 0.76	**2.738 **± 0.14	**13.55 **± 0.27	**15.17 **± 0.45	**24.26 **± 1.64	66.90 ± 2.45
gregarious	**8.03 **± 0.09	**32.28 **± 0.60	**3.027 **± 0.12	**12.24 **± 0.16	**11.65 **± 0.35	**19.90 **± 1.06	68.95 ± 1.94

**Figure 3:**
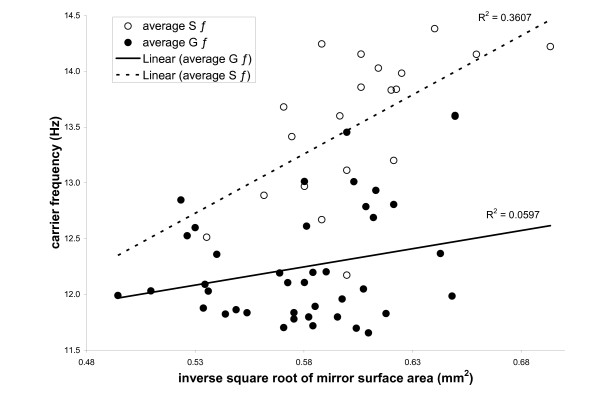
The slope of the regression of C_f _on the inverse square root of mirror surface area was significantly steeper for solitary Mormon crickets.

As expected, carrier frequency (C_*f*_) varied negatively with size (HCW) and mirror surface area, however, there were unexpected aspects to this relationship (figure 3). A full GLM showed that form, mirror surface area, and size all independently influenced C_*f*_, (table 2). Perhaps most interestingly, the relationship between mirror surface area and C_*f *_varied between the forms (figure 3, table 2), which is not predicted if C_*f *_is solely determined by mirror surface area, and suggests that there were effects beyond differences due to size. Solitary males have a higher C_*f *_independent of mirror size, and the slope of the regression of C_*f *_on mirror size is steeper in solitary crickets (figure 3, table 2).

**Table 2:** Predictors of male carrier frequency (C_f_)

Source	df	F	P
Form	1,56	103.65	<<0.001
Mirror surface area	1,56	11.00	0.002
Size (head capsule width)	1,56	5.56	0.022
Form×Mirror surface area	1,56	5.58	0.022

There are two levels of explanation – proximate (mechanistic) and ultimate (evolutionary) – that can address these differences. On a proximate level, the factors that predict C_*f *_may differ between forms. Under the "clockwork cricket" model of stridulation developed from studies of the pure-tone gryllid *Gryllus campestris *[15,16], the stridulatory file represents an escapement mechanism whereby the force (peg strike) causing vibration of the oscillator (harp) is delivered at evenly spaced time intervals due to the incremental slippage and striking of pegs upon the plectrum in much the same way a clock escapement functions. This model predicts that physical properties of the mirror – for example size, shape and tension – predict C_*f *_[15]. In Mormon crickets, it is possible that the tension of the mirror differs between forms, but visual analysis of mirrors did not suggest differences in cuticle density. An alternative model developed from studies of the field cricket *Gryllus bimaculatus *suggests that the volume of the subalar space – a pocket of air enclosed by the raised tegmina during singing – acts as an acoustic resonator for this gryllid [33]. Gryllids may vary the volume of this space, and thus the C_*f *_of their calls, using auditory feedback control [33]. This model has received recent criticism [34]. Our study did not explicitly examine all the factors that may predict C_*f *_in Mormon crickets; however, the fact that the relationship between mirror size and C_*f *_varies between forms suggests that there may be variation between forms in the density or tension of the mirror or the subalar volume.

Other song parameters also varied between forms. Solitary and gregarious Mormon crickets did not differ in the number of pegs on their stridulatory files, but solitary crickets used significantly more pegs during each wing stroke and chirped faster. Thus during an average wing-stroke, solitary individuals struck a greater proportion of their stridulatory pegs on the plectrum. Whether these differences are sufficient to influence female preference is unknown, but fine details of stridulatory peg strikes can influence preference in other tettigoniid species [35].

What ultimately accounts for the morphological and song differences between forms? Gwynne [21] concluded that sex-role reversal caused sexual selection to be stronger on females than males in gregarious populations. Sex-role reversal has been shown to cause changes in song parameters in other tettigoniid species in lab experiments [29] and in the wild [27], and gregarious Mormon crickets are likely to have experienced historically consistent sex-role reversal [21]. Since carrier frequency and chirp rate can convey information about present and past body condition [36,37] and variation in male song can be subject to strong sexual selection [2,3,38-40], it is almost certain that solitary and gregarious cricket populations have experienced differing sexual selection pressures on their calls. However, the forms also broadly correspond with discrete genetic lineages that split during the Pleistocene [31] and song differences can arise via drift during allopatric separation [8]. Drift in allopatry can lead to differences in phenotypic characters important in sexual selection, but it is unlikely to have a directional effect. In Mormon crickets, the direction of almost all differences we uncovered is consistent with the predictions we made based on the previously documented differences [21,22,24] in sexual selection between solitary populations and role-reversed gregarious populations. Gregarious males, as predicted, were more reluctant to sing overall, chirped more slowly independent of temperature, and struck fewer pegs on their plectrum during elytral closing, so we conclude that most of the differences detected here are likely to have arisen in response to sexual selection

### Reluctance to sing

**Figure 4:**
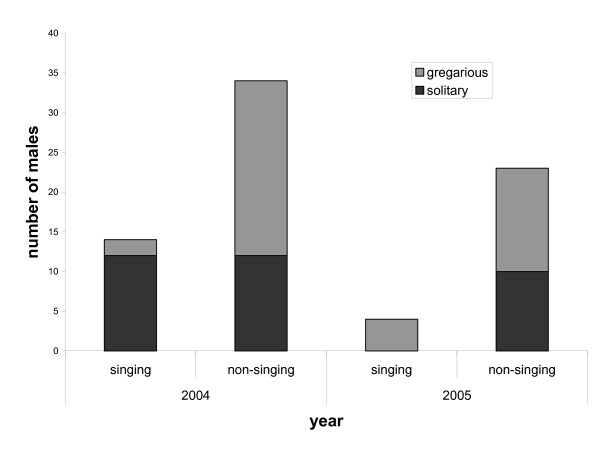
Numbers of singing versus non-singing gregarious and solitary males, by year. Gregarious males were more reluctant to sing overall, although they were less reluctant to sing in 2005. This is the reverse of the trend seen in 2004, and the cause of a significant interaction between year and form (G-test: G_1 _= 10.64, P < 0.05).

Past results [21,22] have indicated that gregarious males should experience diminished sexual selection on song and increased potential to discriminate among females, which we predicted would make them more reluctant to sing. In this study, gregarious males were more reluctant to sing overall (G-test: G_1 _= 4.38, P = 0.036) (figure 4), however while year was not significant as a main effect (G-test: G_1 _= 2.04, NS), temporal variation in this behaviour was inferred from a significant interaction between year and form (G-test: G_1 _= 10.64, P < 0.05). Gregarious males were more reluctant to sing in 2004, but in 2005 solitary males were more reluctant to sing.

Temporal or spatial variation in the number of males and females available for mating [27] could account for this difference, in the sense that phonotaxis to song by natural enemies is likely to cause males to produce less risky calls when many receptive females are available for mating [23]. Numerous studies of ensiferan species have identified acoustically-orienting predators and parasites and adaptations for avoiding them [41-44]. Parasitism risk has been shown to be a significant selection pressure on calling parameters in *Teleogryllus oceanicus*, with crickets in parasitized populations being less likely to sing because parasitoid flies acoustically orient towards singing *T. oceanicus *males [42,45]. Natural enemies of Mormon crickets and other tettigoniids include acoustically orienting vertebrates and possibly ormiine flies [23]. In Mormon crickets, sexual selection pressure on male song may be influenced by an interaction between mate encounter rate by males and the risk of attracting predators and parasites, which are likely to be temporally variable.

### Sexual size dimorphism

**Figure 5:**
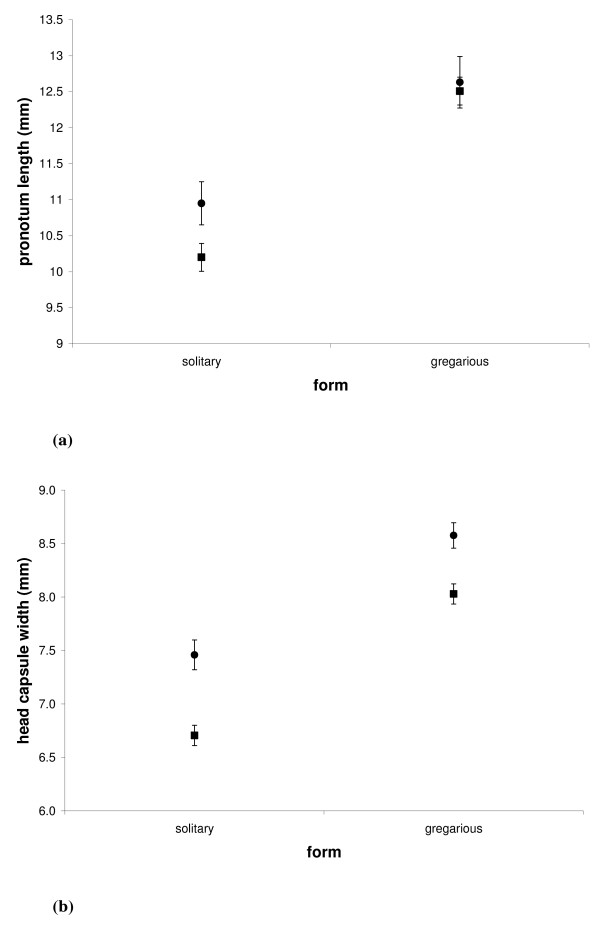
Sexual size dimorphism in solitary and gregarious populations for **(a) **pronotum length and **(b) **head capsule width (HCW). Female means are denoted by circles, and male means by squares, and error bars indicate 95% confidence intervals.

Gwynne [21] described sexual size dimorphism (females larger than males) in gregarious, but not in solitary, populations of Mormon crickets, and attributed this to differences in sexual selection pressure resulting from sex-role reversal. Using the same measure of size (pronotum length), we have shown that in 2005, gregarious individuals are larger than solitary individuals overall (GLM: F_1,81 _= 200.09, P < 0.001) (figure 5a). Females are larger than males in solitary, but not gregarious, populations as indicated by a significant interaction between sexual size dimorphism (inferred from pronotum length) and form (F_1,81 _= 5.01, P = 0.028) (figure 5a). Results using another measure of size (head capsule width) are similar, although sexual size dimorphism was evident in both solitary and gregarious crickets, with females larger than males (GLM: F_1,82 _= 98.82, P << 0.001) (figure 5b). This relationship is constant across both forms, because there is no interaction between sexual size dimorphism (inferred from head capsule width) and form (GLM: F_1,82 _= 2.48, NS) (figure 5b). Variance in size did not differ between solitary and gregarious males and females for either pronotum length (F-test: F = 2.55, NS) or head capsule width (F-test: F = 3.84, NS).

These findings do not concur with Gwynne's [21], that females are larger than males only in gregarious role-reversed populations. Additionally, we found similar variance between sexes and phases, which does not support our hypothesis that gregarious males have experienced relaxed sexual selection. Our results suggest that these measurements of sexual size dimorphism are unlikely to accurately reflect the intensity of sexual selection in these populations. This should ideally be inferred by demonstrating differential mating success directly resulting from variation in the trait of interest [46].

## Conclusion

The behavioural and morphological differences we have found between solitary and gregarious Mormon cricket populations are consistent with the previous discovery of a genetic division that implies a distinct Pleistocene history. Some of these differences are expected given our predictions based on differences in natural history and sexual selection between phases. The unusual variation in the relationship between mirror size and carrier frequency is more surprising, however, and the variation in song frequency in Mormon crickets provides an opportunity for further research into the different models of sound production.

One definition of subspecies is that they should show "concordant distributions of multiple, independent, genetically based traits" [47]. The concept of subspecies has been contentious, yet it remains very important for evolutionary biology and conservation, and comparative studies suggest the taxonomic rank has validity [48]. Two mtDNA clades of Mormon crickets broadly correspond to gregarious and solitary forms [31], and we show here that the two forms are distinct when a broad suite of morphological and song traits are considered together. These animals therefore seem to satisfy subspecies criteria. Solitary and gregarious Mormon crickets from the different clades are capable of successfully mating and transferring spermatophores (pers. obs.), although observations on post-copulatory reproductive isolation are lacking. The existence of a population that is consistently solitary, but phylogenetically and geographically clustered with the gregarious populations [31] suggests that recent or on-going gene flow between the groups occurs, so they are probably not reproductively isolated.

An additional caveat is that our results emphasize the importance of considering the development and causation of divergence in traits used to infer taxonomic distinctions. While differences in most song parameters we studied in Mormon crickets support consistent and strong selection, other behavioural traits like reluctance to sing appear to be very flexible. Species or subspecies boundaries may be obfuscated by plasticity in the traits used to define them, especially song parameters [49], and future studies should carefully address the extent to which such differences are plastic, and their influence on reproductive isolation.

## Methods

### Study populations

Three gregarious populations were sampled in north-western Colorado near Dinosaur National Monument and are coded LE, CO and TM. Solitary populations were sampled at Kelly Flats (KF) and Indian Meadows (IM) in the Poudre Canyon on the eastern slope of the Colorado Rockies. Due to their highly discontinuous distribution, the three gregarious populations were about 300 km away from the two solitary populations.

We used two multivariate analyses to examine differences between the forms. First, a principal components analysis was carried out to see if the solitary and gregarious forms were distinct based on multiple traits (with no prior assumptions about what differences might exist). We tested whether we could combine populations within form for subsequent analyses using an unbalanced nested general linear model (GLM) on the scores from the first principal components axis with form and population as factors. We also carried out a discriminant function analysis with 'form' as the classification variable, using cross-validation, to examine which traits contributed to differences between the solitary and gregarious forms. Both analyses used the following morphological and behavioural measurements: head capsule width (HCW), total surface area of the lower elytron, mirror surface area, number of stridulatory pegs, carrier frequency (C_*f*_), chirp rate and the number of peg strikes per wing stroke.

### Reluctance to sing

'Reluctance to sing' experiments were performed in two separate summers: June and July of 2004 and 2005. In both years, adult solitary males were captured from KF and IM, and adult gregarious males were captured from LE. Subjects were fed an excess of lettuce, yellow sweetclover (*Melilotus officinalis*) and seed heads from grasses, and kept in same-sex cages with approximately 15 individuals for at least three days, to ensure that males who may have mated previously replenished their accessory glands, which produce the complex spermatophore.

In 2004, reluctance to sing was assessed by placing individual males in a cylindrical mesh cage (approx. 30 cm in diameter and 45 cm long) placed half in sunlight and half in shade in an enclosed chamber. In these experiments, males always moved to the sunny portion of the cage regardless of whether they sang or not (pers. obs.). Mormon crickets can sing continuously for many consecutive minutes, or song can be broken into shorter bouts of singing. Individual chirps are produced during the closing motion of the wings and each peg strike produces highly damped oscillations of the mirror (Figure 1). Calling activity was recorded for 10 minutes using a Sennheiser ME 66 directional microphone with a K6 powering module and a sampling rate of 96,000 samples per second. The microphone had a nominal frequency response of 50 Hz to 20 kHz, so frequencies above 20 kHz were unlikely to be obtained (carrier frequency of Mormon cricket songs in this study ranged from 11.6 kHz to 14.4 kHz). Recordings were made digitally in the field using Sony Sound Forge 7.0a software (Sony Pictures Digital Inc. 2003) installed on a laptop computer. Air temperature in the shade was noted using a thermocouple accurate to the nearest tenth of a degree Centigrade. We used 24 solitary males and 24 gregarious males, chosen randomly, and all trials were run between 8:30 am and 1:00 pm when Mormon crickets are most active. In 2005, the experiment was performed in the field as close to the original population as possible but far enough away to be out of hearing range of other singing crickets (approx. 1 km). The mesh cage (approximately 10 cm in diameter and 25 cm long) was placed in a clump of sagebrush, and the rest of the experiment was as before. Seventeen gregarious males and 10 solitary males were used. We tested whether the incidence of singing versus non-singing males differed between solitary and gregarious populations and between years using a 3-factor G test [50].

### In-situ recordings

In a separate experiment to test the relationship between carrier frequency and mirror size, gregarious males were recorded in the field from LE, TM and CO (n = 16, n = 8 and n = 16) and solitary males were recorded from IM and KF (n = 10 and n = 11) during June and July of 2005 as before. Care was taken not to disturb the crickets while they were being recorded. The temperature at the time of recording was noted, and after each male stopped singing it was captured and preserved in ethanol. Carrier frequency (C_*f*_) was determined by analysing five randomly chosen one-quarter second samples per individual using a Fast-Fourier Transform (FFT) of size 32,768 with a Blackman-Harris smoothing window, and the average was used in analyses.

To determine what factors predicted C_*f*_, we used a full GLM with temperature, head capsule width (HCW), form and mirror surface area as the independent variables. In tettigonids, the length of the mirror frame has been suggested to determine carrier frequency [17]; however, Mormon cricket mirrors are unusual in that they are almost perfectly circular. The frequency produced by a circular membrane is expected to vary according to the following equation:

*f *= [k/D]*[√(T/σ)]     (1)

where k is a constant, D is diameter of the membrane, T is tension of the membrane and σ is the density of the membrane [51]. If T and σ remain constant, D can be expressed in terms of surface area (S_a_) as √(4S_a_/π) and substituting into (1) gives:

*f *= b/[√(S_a_)]     (2)

where b is constant and S_a _is mirror surface area. Given this expected inverse square root relationship between C_*f  *_and mirror surface area, we transformed mirror surface area by inverting then taking the square root of the variable before adding it as a factor to the linear model. We included two interaction terms; one between phase and HCW, and the other between phase and mirror surface area. The final model was reduced to form, mirror surface area, HCW and a form×mirror surface area interaction term.

For each recording, we counted the average number of wing-strokes per second and the average number of peg strikes against the plectrum within each wing stroke. Given that chirp rate in other tettigoniines has been shown to be a linear function of temperature [52], we included temperature as a factor in separate GLMs testing whether chirp rate and average peg strikes per chirp differed between forms.

### Morphology

We measured head capsule width (HCW), pronotum length, number of stridulatory pegs, the total surface area of the lower elytron and mirror surface area for all males. A small sample of 12 gregarious (LE) and 12 solitary (KF and IM) females were also measured for HCW and pronotum length to assess any sexual dimorphism, as described by Gwynne [21], who found that females were larger than males in gregarious populations but not in solitary populations. We assessed this using a GLM with sex, form and two interaction terms as factors. We also tested for any differences in variance in size between sexes and forms using an F-test.

## Authors' contributions

NWB participated in the design of field studies, carried them out, analysed the data and drafted the manuscript. DTG participated in the design and execution of field studies and helped draft the manuscript. WVB participated in the execution of field studies and analysis of songs. MGR participated in the design of the study, statistical analyses and helped draft the manuscript.

## References

[B1] West-Eberhard MJ (1983). Sexual selection, social competition, and speciation. Q Rev Biol.

[B2] Andersson M (1994). Sexual selection.

[B3] Henry CS (1994). Singing and cryptic speciation in insects. Trends Ecol Evol.

[B4] Mandelson TC, Shaw KL (2005). Rapid speciation in an arthropod. Nature.

[B5] Coyne JA, Orr HA (1989). Patterns of speciation in *Drosophila*. Evolution.

[B6] Roff DA, Mosseau TA, Howard DJ (1999). Variation in geneticarchitecture of calling song among populations of *Allonemobius socius*, *A. fasciatus*, and a hybrid population: drift or selection?. Evolution.

[B7] Gleason JM, Ritchie MG (1998). Evolution of courtship song and reproductive isolation in the *Drosophila willistoni *species complex: do sexual signals diverge the most quickly?. Evolution.

[B8] Tregenza T, Pritchard VL, Butlin RK (2000). The origins of premating reproductive isolation: testing hypotheses in the grasshopper *Chorthippus parallelus*. Evolution.

[B9] Butlin RK, Ritchie MG, Slater PJB, Halliday TJ (1994). Behavior and speciation. Behavior and Evolution.

[B10] Ritchie MG, Racey SN, Gleason JM, Wolff K (1997). Variability of the bushcricket *Ephippiger ephippiger*: RAPDs and song races. Heredity.

[B11] Bennet-Clark HC, Huber F, Moore TE, Loher W (1989). Songs and the physics of sound production. Cricket behavior and neurobiology.

[B12] Ewing AW (1989). Arthropod bioacoustics; neurobiology and behavior.

[B13] Greenfield MD, Bailey WJ, Rentz DCF (1990). Evolution of acoustic communication in the genus *Neoconocephalus*: discontinuous songs, synchrony and interspecific interactions. The Tettigoniidae: biology, systematics and evolution.

[B14] Montealegre-Z F, Morris GK (1999). Songs and systematics of some tettigoniidae from Colombia and Ecuador I. Pseudophyllinae (Orthoptera). J Orthop Res.

[B15] Elliot CJH, Koch UT (1985). The clockwork crickets. Naturwissenschaften.

[B16] Koch UT, Elliot CJH, Schaffner K-H, Kleindienst H-U (1988). The mechanics of stridulation of the cricket *Gryllus campestris*. J Comp Physiol A.

[B17] Sales G, Pye D (1974). Ultrasonic communication in animals.

[B18] Cowan FT (1990). The Mormon cricket story. Agric Exp Station Special Report.

[B19] Lorch PD, Sword GA, Gwynne DT, Anderson GL (2005). Radiotelemetry reveals differences in individual movement patterns between outbreak and non-outbreak Mormon cricket populations. Ecol Ent.

[B20] Sword GA, Lorch PD, Gwynne DT (2005). Migratory bands give crickets protection. Nature.

[B21] Gwynne DT (1984). Sexual selection and sexual differences in Mormon crickets (Orthoptera: Tettigoniidae, *Anabrus simplex*). Evolution.

[B22] Gwynne DT (1981). Sexual difference theory: Mormon crickets show role reversal in mate choice. Science.

[B23] Gwynne DT (2001). Katydids and bush-crickets.

[B24] Gwynne DT (1993). Food quality controls sexual selection in Mormon crickets by altering male mating investment. Ecology.

[B25] Simpson SJ, Sword GA, Lorch PD, Couzin ID (2006). Cannibal crickets on a forced march for protein and salt. Proc Natl Acad Sci USA.

[B26] Gwynne DT (1985). Role reversal in katydids: Habitat influences reproductive behaviour (Orthoptera: Tettigoniidae, *Metaballus *species). Behav Ecol Sociobiol.

[B27] Gwynne DT, Bailey WJ, Annells A (1998). The sex in short supply for matings varies over small spatial scales in a katydid (*Kawanaphila nartee*, Orthoptera: Tettigoniidae). Behav Ecol Sociobiol.

[B28] Hissman K (1990). Strategies of mate finding in the European field cricket (*Gryllus campestris*) at different population densities: a field study. Ecol Entomol.

[B29] Ritchie MG, Sunter D, Hockham LR (1998). Behavioral components of sex role reversal in the Tettigoniid bushcricket *Ephippiger ephippiger*. J Insect Behav.

[B30] MacVean CM, Capinera JL (1987). Ecology and management of the Mormon cricket, *Anabrus simplex *Haldeman. Integrated Pest Management on Rangeland: A Shortgrass Prairie Perspective.

[B31] Bailey NW, Gwynne DT, Ritchie MG (2005). Are solitary and gregarious Mormon crickets (*Anabrus simplex*, Orthoptera, Tettigoniidae) genetically distinct?. Heredity.

[B32] Zuk M, Kolluru GR (1998). Exploitation of sexual signals by predators and parasitoids. Q Rev Biol.

[B33] Stephen RO, Hartley JC (1995). Sound production in crickets. J Exp Biol.

[B34] Prestwich KN, Lenihan KM, Martin DM (2000). The control of carrier frequency in cricket calls: a refutation of the subalar-tegminal resonance/auditory feedback model. J Exp Biol.

[B35] Ritchie MG, Couzin ID, Snedden WA (1995). What's in a song? Female bushcrickets discriminate against the song of older males. Proc R Soc Lond B.

[B36] Scheuber H, Jacot A, Brinkhof MWG (2003). Condition dependence of a multicomponent sexual signal in the field cricket *Gryllus campestris*. Anim Behav.

[B37] Scheuber H, Jacot A, Brinkhof MWG (2003). The effect of past condition on a multicomponent sexual signal. Proc R Soc Lond B.

[B38] Simmons LW (1995). Correlates of male quality in the field cricket *Gryllus campestris *L.: age, size, and symmetry determine pairing success in field populations. Behav Ecol.

[B39] Simmons LW, Ritchie MG (1996). Symmetry in the songs of crickets. Proc R Soc Lond B.

[B40] Shaw KL, Herlihy DP (2000). Acoustic preference functions andsong variability in the Hawaiian cricket *Laupala cerasina*. Proc R Soc Lond B.

[B41] Belwood JJ, Morris GK (1987). Bat predation and its influence on calling behavior in neotropical katydids. Science.

[B42] Zuk M, Simmons LW, Cupp L (1993). Calling characteristics of parasitized and unparasitized populations of the field cricket *Teleogryllus oceanicus*. Behav Ecol Sociobiol.

[B43] Römer H, Bailey W (1998). Strategies for hearing in noise: peripheral control over auditory sensitivity in the bushcricket *Sciarasaga quadrata *(Austrosaginae: Tettigoniidae). J Exp Biol.

[B44] Schul J, Patterson AC (2003). What determines the tuning of hearing organs and the frequency of calls? A comparative study in the katydid genus *Neoconocephalus *(Orthoptera, Tettigoniidae). J Exp Biol.

[B45] Zuk M, Rotenberry JT, Simmons LW (1998). Calling songs of field crickets (*Teleogryllus oceanicus*) with and without phonotactic parasitoid infection. Evolution.

[B46] Panhuis TM, Butlin R, Zuk M, Tregenza T (2001). Sexual selection and speciation. Trends Ecol Evol.

[B47] Avise JC, Ball RJM, Futuyma D, Antonovics J (1990). Principles of genealogical concordance in species concepts and biological taxonomy. Oxford Surveys in Evolutionary Biology.

[B48] Phillmore AB, Owens IPF (2006). Are subspecies useful in evolutionary and conservation biology?. Proc R Soc Lond B.

[B49] Olvido AE, Mousseau TA (1995). Effect of rearing environment on calling-song plasticity in the striped ground cricket. Evolution.

[B50] Sokal RR, Rohlf FJ (1969). Biometry.

[B51] Sears FW, Zemansky MW (1964). University Physics Third edition complete.

[B52] Walker TJ (1974). Effects of temperature, humidity, and age on stridulatory rates in *Atlanticus *spp. (Orthoptera: Tettigoniidae: Decticinae). Ann Ent Soc Amer.

